# Use of the probiotic *Lactobacillus plantarum *299 to reduce pathogenic bacteria in the oropharynx of intubated patients: a randomised controlled open pilot study

**DOI:** 10.1186/cc7109

**Published:** 2008-11-06

**Authors:** Bengt Klarin, Göran Molin, Bengt Jeppsson, Anders Larsson

**Affiliations:** 1 Department of Anaesthesiology and Intensive Care, University Hospital, SE-221 85 Lund, Sweden; 2 Applied Nutrition and Food Chemistry, Lund University, Box 117, SE-221 00 Lund, Sweden; 3 Department of Surgery, University Hospital, SE-205 02 Malmö, Sweden; 4 Department of Anaesthesiology and Intensive Care, Aalborg Hospital, Århus University Hospitals, DK-9000 Aalborg, Denmark

## Abstract

**Introduction:**

Ventilator-associated pneumonia (VAP) is usually caused by aspiration of pathogenic bacteria from the oropharynx. Oral decontamination with antiseptics, such as chlorhexidine (CHX) or antibiotics, has been used as prophylaxis against this complication. We hypothesised that the probiotic bacteria *Lactobacillus plantarum *299 (Lp299) would be as efficient as CHX in reducing the pathogenic bacterial load in the oropharynx of tracheally intubated, mechanically ventilated, critically ill patients.

**Methods:**

Fifty critically ill patients on mechanical ventilation were randomised to either oral mechanical cleansing followed by washing with 0.1% CHX solution or to the same cleansing procedure followed by oral application of an emulsion of Lp299. Samples for microbiological analyses were taken from the oropharynx and trachea at inclusion and at defined intervals thereafter.

**Results:**

Potentially pathogenic bacteria that were not present at inclusion were identified in oropharyngeal samples from eight of the patients treated with Lp299 and 13 of those treated with CHX (p = 0.13). Analysis of tracheal samples yielded similar results. Lp299 was recovered from the oropharynx of all patients in the Lp299 group.

**Conclusions:**

In this pilot study, we found no difference between the effect of Lp299 and CHX used in oral care procedures, when we examined the effects of those agents on colonisation of potentially pathogenic bacteria in the oropharynx of intubated, mechanically ventilated patients.

## Introduction

Ventilator-associated pneumonia (VAP) is a common complication in intubated, mechanically ventilated patients in intensive care units (ICUs). VAP is connected to longer ICU and hospital stays, additional costs and high mortality, and the risk of developing this condition increases by 1% with each additional day of mechanical ventilation [1,2].

The major cause of VAP is aspiration of either microorganisms from the oropharynx or fragments of biofilms from the endotracheal tube. Formation of such biofilms can be delayed, but not prevented, by the use of tubes with special coatings [3]. Selective decontamination using antibiotics in the oral cavity alone [4-6] or throughout the gastrointestinal (GI) tract [7,8], has been shown to lower the incidence of VAP and reduce mortality. However, the use of such procedures is limited due to the risk of bacteria developing resistance to the antibiotics [9,10]. In recent meta-analyses, it was concluded that oral decontamination with chlorhexidine (CHX) could prevent VAP [11], but that strategy does not reduce the time on the ventilator, the length of stay (LOS) in the ICU or rates of mortality [12]. Thus, there is a need for alternative approaches to lower the oropharyngeal load of pathogenic microorganisms as a means of decreasing the risk of VAP.

For decades, probiotics have been given enterally to improve the microbiotic flora in the GI tract. However, in recent years orally administered probiotics have also been shown to reduce the number of bacteria and yeast in biofilms on vocal prostheses [13,14]. Therefore, we hypothesised that swabbing the oral mucosa with probiotics would be an effective (and microbiologically attractive) method of reducing pathogenic oral microorganisms in tracheally intubated, mechanically ventilated, critically ill patients.

The primary aim of the present pilot study was to evaluate the feasibility and safety of an oral care procedure using the probiotic *Lactobacillus plantarum *299 (Lp299) (DSM 6595) in this patient category. Like the genomically closely related strain *L plantarum *299v (DSM 9843), Lp299 has been shown to adhere to the mucosa throughout the GI tract [15-17]. Another objective of this preliminary investigation was to obtain an estimate of the number of patients needed for a definitive study examining the effectiveness of oral Lp299 in reducing the incidence of VAP.

## Materials and methods

The study was approved by the Human Ethics Committee of Lund University and was performed in compliance with the Helsinki Declaration. Good clinical practice and the International Conference on Harmonisation Guidance were applied and the investigation was carried out in the ICU of the Department of Anaesthesiology and Intensive Care, University Hospital, Lund, Sweden. Informed consent was obtained from the patients or their next of kin. Further consent was not obtained from patients as they had recovered, as this was not required by the Human Ethics Committee.

The patients were randomised into groups of 10 to receive either the department's standard oral treatment (the control group) or the study treatment with Lp299 (the Lp group). The day of inclusion was designated day 1. To be included in the study, patients had to fulfil the following criteria: 18 years of age or older; critically ill with an anticipated need for mechanical ventilation of at least 24 hours; not moribund; not suffering from pneumonia at admission; no fractures in the facial skeleton or the base of the skull; no oral ulcers; not immune deficient; not a carrier of HIV or viral hepatitis.

After screening, patients were included when ventilation and circulation had been stabilised and before the first oral care procedure. Oral care was performed twice a day. The control group was treated according to the department's standard protocol: dental prostheses were removed; secretions were removed by suction; teeth were brushed using toothpaste (Zendium, Opus Health Care, Malmö, Sweden); all mucosal surfaces were cleansed with swabs that had been moistened with a 1 mg/ml CHX solution (Hexident, Ipex, Solna, Sweden). In the Lp group the initial mechanical steps were the same as in the control group, but the subsequent cleansing was instead performed with gauze swabs soaked in carbonated bottled water, after which Lp299 was applied to the mucosal surface of the oral cavity. This was performed using two gauze swabs (one for each side of the oral cavity), which had been allowed to absorb 10 ml of a solution containing a total of 10^10 ^colony-forming units (CFU) of Lp299. Excess suspension was not removed. In both groups, when necessary between the oral care procedures, secretions were removed by suctioning and gauze swabs moistened with carbonated bottled water were used to wipe off remaining secretions.

Cultures were taken from the oropharynx and from the trachea at inclusion. Sampling was repeated before the oral care procedures on days 2, 3, 5, 7, 10, 14 and 21 in patients that were still mechanically ventilated. If a patient was extubated on a non-culture day, cultures were taken before the extubation. One set of cultures was analysed according to normal routines at the Department of Clinical Microbiology, University Hospital. Another set was sent blinded to the research laboratory at Probi AB, Lund, Sweden for identification and quantification of total CFU of lactobacilli and identification of Lp299. Viable counts of all lactobacilli were obtained on Rogosa agar (Oxoid, Basingstoke, Hampshire, England) incubated anaerobically at 37°C for three days. Colonies suspected to be Lp299 (large, creamy white-yellowish and somewhat irregular in shape) were selected and identified by randomly amplified polymorphic DNA typing [18].

The patients were placed in a semi-recumbent position and were ventilated in pressure control or pressure support mode by a Servo ventilator (Maquet AB, Sweden) via a heat moisture exchange filter (Barrierbac "S", Mallinckrodt DAR, Mirandola, Italy). A closed suction system (TRACH-Care 72, Ballard Medical Products, Draper, UT, USA) was used. The patients inhaled 2.5 mg salbutamol (GlaxoSmithKline, Solna, Sweden) and 0.5 mg ipratropium (Boehringer Ingelheim, Stockholm, Sweden) every six hours.

Chest radiographs were obtained after tracheal intubation and thereafter when clinically indicated. Lung function was assessed using the Lung Injury Score (LIS) [19]. Blood gases were obtained at least three times a day and were analysed at the ICU. Samples for white blood cell (WBC) counts and C-reactive protein (CRP) were collected daily and analysed at the hospital clinical chemistry laboratory.

Enteral nutrition was started and increased according to the department's protocol. The amount of enteral formula given and the total volume of other enterally administered fluids were recorded. All patients received intravenous ezomprazol (Astra Zeneca, Södertälje, Sweden) as stress ulcer prophylaxis from admission until enteral nutrition was fully established (ie, for three to four days).

The study was neither intended nor powered for assessment of differences in the frequency of VAP. However, it was aimed at obtaining a basis for estimating the number of patients needed for a larger investigation in which VAP also constitutes a parameter. The following criteria were used to identify VAP: a new, persistent or progressive infiltrate on chest radiograph combined with at least three of the other four criteria; a purulent tracheal aspirate; positive culture of tracheal aspirates occurring after 48 hours of mechanical ventilation; rectal or urine bladder temperature higher than 38.0°C or less than 35.5°C; WBC count more than 12 or less than 3 [4,20].

### Statistics

Because no previous investigation has examined the effect of probiotics in this context, we estimated that 20 patients in each group would be sufficient to assess the safety, important positive effects and possible side effects, and to give an indication of the number of patients that would be needed in a definitive study. Statistical methods were chosen after consulting a biostatistician, and the statistical analyses were performed using Statistica 6.0 (StatSoft, Tulsa, OK, USA). Student's t-test was used for the daily comparisons (days1 to 9) of the parameters. Fisher's exact test was employed to compare the results of microbiological cultures. p < 0.05 was considered significant.

## Results

After screening, 50 patients were included in the study. Consent was withdrawn by two patients and another three were transferred to other ICUs shortly after inclusion. For one patient in the control group, samples were obtained only at inclusion. Altogether, 23 patients in the Lp group and 21 in the control group completed the study.

All patients were orotracheally intubated. Two in each group were reintubated, and two in the Lp group and one in the control group were tracheotomised (on days 3, 16 and 3, respectively). The proportion of patients receiving enteric nutrition and the volumes given were similar in the two groups. The patients in both groups were treated with antibiotics at the discretion of the attending physician and changes were made in compliance with culture results. Cefuroxime was the most common antibiotic used in both groups, followed by imipenem. Three patients in each group received piperacillin/tazobactam, and other antibiotics or combinations were administered to some patients in each of the two groups. Three patients did not receive any antibiotics at admission, and one of those three was not treated with antibiotics during the stay in the ICU. Ten patients in each group received corticosteroids for one or more days.

As indicated in Table 1, there were no significant differences in age or gender between the groups. Also, the admission diagnoses were similar in the two groups, as were the Acute Pathophysiology and Chronic Health Evaluation (APACHE) II scores. Some differences were found in the Sequential Organ Failure Assessment (SOFA) scores in favour of the Lp patients (data not shown). The two groups did not differ significantly with regard to the number of ventilator days, LOS, or ICU or in-hospital mortality (Table 1). No deaths were caused by respiratory complications and no additional deaths occurred within six months.

**Table 1:** Patient characteristics and admission diagnosis

	**Lp299 group**	**Control group**
**Age**	70 (20 to 87)	70 (43 to 81)
**Sex M/F**	13/10	9/12
**APACHE II score**	22 (11 to 39)	27(9 to 37)
**ICU mortality**	5/23	4/21
**In-hospital mortality**	5/23	6/21
**ICU stay (days)**	7.7 (1.3 to 26.1)	6.6 (1.3 to 16.0)
**Ventilator days**	5.8 (1.0 to 23.8)	4.3 (1.0 to 15.2

**Diagnosis at admission**	**Lp299 group**	**Control group**

**Sepsis, septicaemia**	6	5
**Other infections**	2	1
**Cardiological: arrests and insufficiencies**	5	4
**Respiratory insufficiencies**	3	5
**Abdominal**	1	2
**Vascular (emergency aneurysms)**	0	3
**Trauma**	3	0
**Other**	3	1

Data are presented as median (range) except for sex and death rates.

Differences are not significant.

APACHE = Acute Pathophysiology and Chronic Health Evaluation; ICU = intensive care units; Lp299 = *Lactobacillus plantarum *299.

No differences in WBC counts were found between the groups. Furthermore, the groups did not differ with regard to changes in CRP, although the absolute values were higher for the controls on day 3.

**Figure 1:**
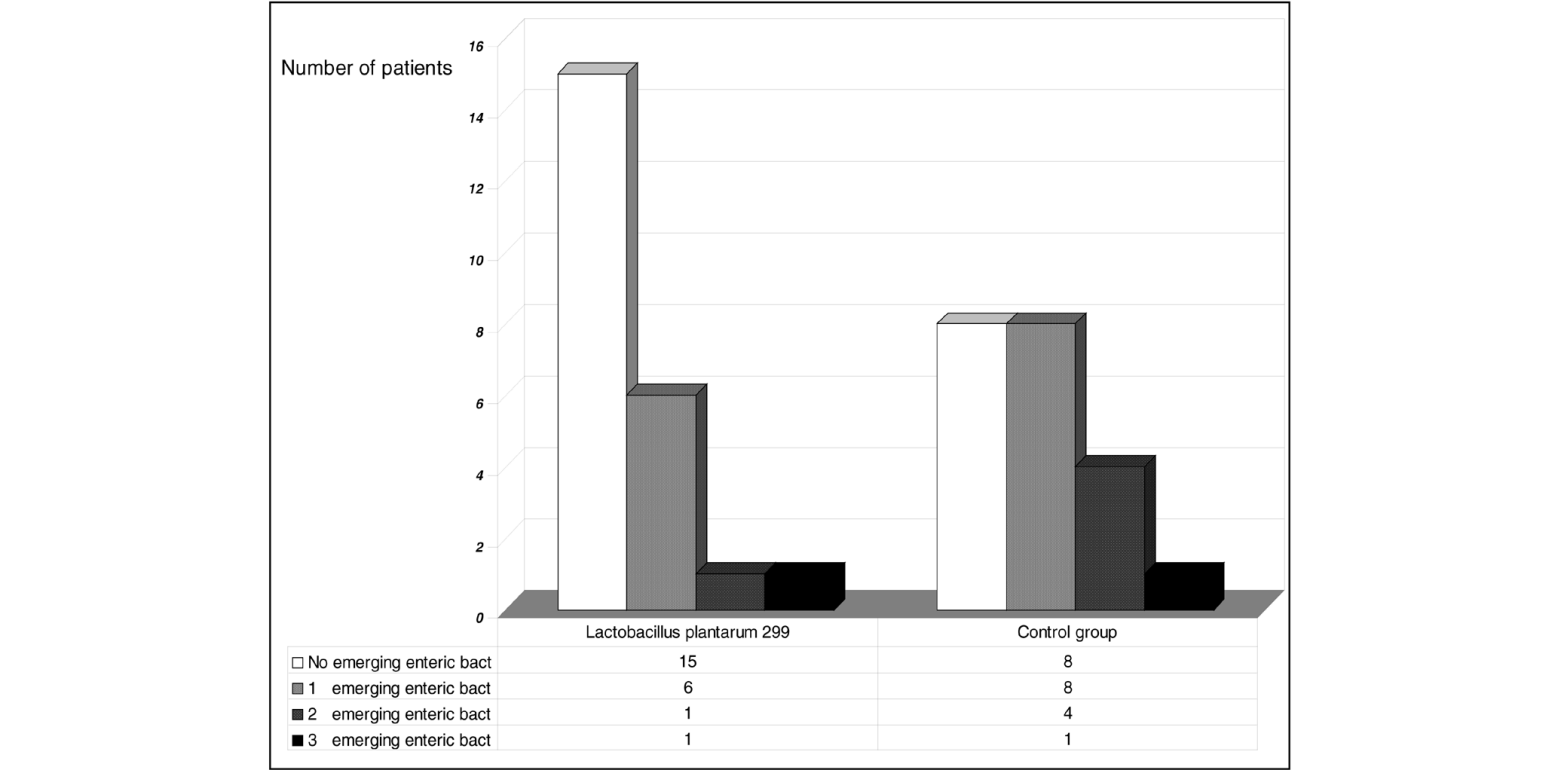
**Results of oropharyngeal cultures**. Number of patients with and without emerging enteric bacteria, not identified at inclusion. No new enteric species (ie, taxa not found at inclusion) appeared in 65% (15 of 23) of the patients in the Lp299 group compared with 38% (8 of 21) in the control group.

**Figure 2:**
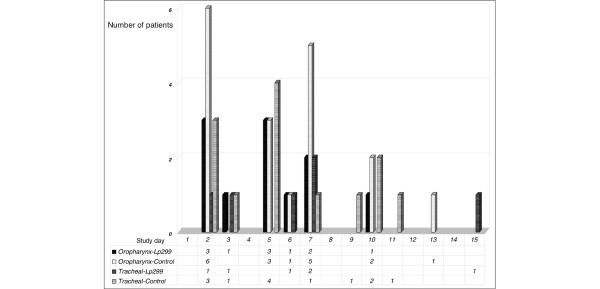
**Distribution of the findings of emerging enteric bacteria**. On the first days of ICU care, identified emerging enteric species were twice as many in the control patients. Despite a gradual decrease in the number of patients remaining in the study (similar in both groups), new cases of tracheal infection appeared in the latter part of the study period, primarily in the control group.

No significant differences between the two groups were found when considering microbiological findings of the oropharyngeal and tracheal samples taken at inclusion. The same species were identified in samples from both the oropharynx and the trachea of six Lp patients and three controls. Subsequent oropharyngeal samples from eight Lp patients and from 13 controls contained enteric species that had not been present in the inclusion samples from those patients (p = 0.13) (Table 2). Two or three emerging species (enterococci species and enterobacteriaceae species) were found in two patients in the Lp group and seven control patients (Figure 1). Culture analysis of the tracheal samples identified emerging species in seven Lp patients and nine controls. Other comparisons of the culture results were similar. Figure 2 shows the distribution of the positive cultures according to study day and sampling site.

**Table 2:** Number of positive findings of bacteria species at inclusion and in subsequent samples

	**Throat samples**				**Tracheal secretions**			
	
**Species**	**Inclusion**		**Subsequent**		**Inclusion**		**Subsequent**	
	
	**Lp**	**C**	**Lp**	**C**	**Lp**	**C**	**Lp**	**C**
1 *Haemophilus influenzae*	1	0	0	0	1	2	1	0
2 *Moraxella catarrhalis*	0	0	0	0	0	1	1	0
3 *Beta-Streptococcus group G*	1	0	1	0	0	0	0	0
4 *Streptococcus pneumoniae*	1	0	1	0	2	0	0	0
5 *Streptococcus pyogenes*	0	1	0	0	0	1	0	0

1–5 Airway bacteria	3	1	2	0	3	4	2	0

6 *Staphylococcus aureus*	6	2	1	0	3	0	2	0

7 Citrobacter species	0	0	1	0	0	0	1	0
8 *Escherichia coli*	1	2	1	2	1	1	0	1
9 *Enterobacter aerogenes*	1	2	0	0	0	0	0	1
10 *Enterobacter cloacae*	1	1	0	2	1	1	0	1
11 *Hafnia alvei*	0	0	0	1	0	0	0	0
12 *Klebsiella oxytoca*	0	0	1	0	0	1	0	1
13 *Morganella morgani*	0	0	0	1	0	0	0	0
14 *Proteus mirabilis*	0	0	1	1	1	0	0	1
15 *Proteus vulgaris*	0	0	1	0	0	0	1	0
16 *Pseudomonas aeruginosa*	0	0	1	2	2	1	0	1
17 Pseudomonas species	0	0	1	1	0	0	2	0
18 *Serratia marcescens*	0	0	0	0	0	0	1	0
19 Serratia species	0	0	0	0	1	0	0	0
20 *Stenotrophomonas maltophilia*	1	0	0	2	0	0	1	1
21 *Streptococcus agalactiae*	1	1	0	0	0	0	0	1
22 *Enterococcus faecalis*	0	0	3	3	0	0	1	2
23*Enterococcus faecium*	1	0	1	2	0	0	0	2

7–23 Enteric bacteria	6	6	11	17	6	4	7	12

24 *Candida albicans*	5	4	5	9	3	7	5	5
25 *Candida parapsilosis*	0	0	1	0	0	0	1	0
*26 Candida tropicalis*	0	0	0	0	0	1	0	0

24–26 Fungi	5	4	6	9	3	8	6	5

Only the first sample in which the species was identified is included in the presented data.

All the isolated *Staphylococcus aureus *strains were non-*MRSA*.

No significant differences were found between the two groups.

Lp = patients treated with *Lactobacillus plantarum *299; C = control patients treated with chlorhexidine.

Lp299 was found in the oropharyngeal samples from all of the patients in the Lp group (21 of 23 patients on day 2). In addition, Lp299 was identified in the tracheal secretion samples from 13 of the patients in the Lp group (56%), and enteric bacteria were also found in six of those subjects. Five patients in the Lp group died in the ICU, and Lp299 was identified in the tracheal samples from one of those individuals, whereas no enteric bacteria were recovered from the trachea of any of those five patients.

Considering patients in both groups, a comparison of those with positive findings and those with negative findings in cultures of tracheal secretions (results reported by the microbiology laboratory) indicated a significantly lower number of ventilator days (p < 0.001) in the non-colonised subjects. VAP was identified in one patient in the Lp299 group and in three patients in the CHX group.

## Discussion

This pilot study shows that it is feasible and safe to use Lp299 as an adjunct in the oral care of intubated patients. When we compared patients subjected to an Lp299-based oral care procedure with those who underwent the standard CHX-based oral treatment used at the department, we did not find any significant difference in the incidence of emerging, potentially pathogenic bacteria in the oropharynx or trachea. The emerging bacteria were, as expected, mainly Gram-negative species.

The use of CHX in oral care procedures is considered to be an effective method to reduce pathogens in the oropharynx and to prevent VAP [11,12]. Aspiration of pathogenic bacteria constitutes the main cause of VAP, and thus reducing the occurrence of such microorganisms in the oropharynx should lower the rate of VAP. In our study, pathogenic enteric bacteria appeared in fewer the patients in the Lp299 group (38%) than in the CHX group (65%). This indicates that Lp299 might be able to lower the rate of infection with such harmful microbes and in turn lead to fewer cases of VAP. As anticipated, the difference in the incidence of VAP between the treatment groups in our study (one case in the Lp299 group and three in the CHX group) was inconclusive.

It should be mentioned that there are some common side effects associated with CHX use in oral care, including discoloration of the teeth, a burning sensation on the tongue and irritation of the mucosa [21,22]. More serious but rare adverse effects are local allergic reactions in the mouth and throat. Of particular importance is that CHX shows little activity against Gram-negative bacteria [23]. Moreover, it is diluted and inactivated by saliva [24], and since bacteria can be resistant to CHX, a low concentration (which will regularly occur between oral care treatments) represents an additional risk of selection and emergence of resistant strains. What is even more alarming is that bacteria strains that are not susceptible to common antibiotics, such as methicillin-resistant *Staphylococcus aureus *(MRSA) also often carry genes for resistance to CHX [25]. *L plantarum *strains are genetically stabile and are not likely to incorporate resistance genes or plasmids or to transfer genetic material, characteristics that are related to their inherent resistance to certain antibiotics and to other species. Consequently *L plantarum *does not contribute to the development of antibiotic-resistant strains. In humans, lactobacilli colonise the oropharynx soon after birth, and thereafter constitute part of the normal oropharyngeal flora and, accordingly, these bacteria will enter the lower respiratory tract whenever an aspiration occurs. Other strains of lactobacillus than Lp299 have in immunocompromised patients been associated with severe infections such as endocarditis [26-28].

A limitation of our study is that we did not perform surveillance blood cultures, although the Lp299 aspirated did not produce any detectable infiltrates indicating pneumonia or bacteraemia. Furthermore, aspiration of Lp299 alone did not influence the oxygenation index, LOS or days of mechanical ventilation. Notably, the genomically closely related *L plantarum *299v, has been found to be safe in an animal model of endocarditis [29]. In that study, *L plantarum *299v could not be detected in the blood or heart of the laboratory animals, nor on implanted catheters 96 hours after intravenous injection of the bacteria. Both Lp299 and *L plantarum *299v have also been proven safe for enteral use in the ICU setting [16,30-34].

Furthermore, except for the calculated risk of aspiration, so far we have not seen any side effects of using Lp299 as an alternative in oral care. It may be more effective to add other probiotic bacteria to the treatment suspension, but at present we do not consider that approach to be safe, because it was recently found that enteral administration of a mixture of six strains of probiotics (none of them *L plantarum*) was associated with increased mortality in patients with severe pancreatitis [35]. In contrast to those results, studies of *L plantarum *299 and 299v given enterally to critically ill patients have not revealed any adverse effects of those strains [16,30-34]. Also, although we did not remove excess Lp299 suspension after the oral care procedure, some of the bacteria must have reached the GI tract, where they probably had a positive influence on the microflora. A combination of enteral and oral treatments would probably have a greater impact on the oral flora, because if any gastric content is regurgitated, it is likely to have a lower content of potentially pathogenic bacteria.

The oral care procedure in the present study was performed twice a day, which seems to correspond to the protocols in use in many ICUs [11], although it is plausible that even better results can be obtained by treating more frequently, as performed by Koeman and colleagues [36]. According to most of the relevant studies in the literature, as well as a meta-analysis [11,12] different preparations and concentrations of CHX have been effective in reducing the incidence of VAP.

Lactobacillus species can be detected in interdental spaces, plaques and carious lesions [37], but we have found no data in the literature that seem to suggest a link between lactobacilli and initiation of caries. On the contrary, two Finnish studies have shown improved dental status and lowered counts of *Streptococcus mutans *in school children who consumed milk or cheese containing *Lactobacillus rhamnosus *GG [38,39]. Furthermore, in an investigation of different species of lactobacillus, it was observed that *L plantarum *strains had the most pronounced antimicrobial effect on *S mutans*, and they were also highly efficacious against other pathogens that are frequently found in periodontal disease [40].

The present results indicate that Lp299 might be used as a component of oral care in intubated ICU patients. Besides offering a promising alternative to antiseptics like CHX, a probiotic that adheres to the oral mucosa will be able to counteract potentially pathogenic bacteria 24 hours a day, which is superior to the fairly short-term effect of orally applied chemical agents.

Clearly, it is also important to point out that the findings of this pilot study must be interpreted with great caution, and the trends indicated by the data must and will be further examined in a larger investigation. Nevertheless, our main objectives have been met, because we found that Lp299 did become established in the oral cavity, it had no apparent adverse effects and the results provide a basis for calculating the number of patients needed to test the trends observed in the planned definitive study.

## Conclusion

Based on the results of this pilot study, we conclude that the probiotic bacterium Lp299 constitutes a feasible and safe agent for oral care. Also, it seems that Lp299 is as effective as CHX in mitigating colonisation with pathogenic bacteria in the oropharynx of intubated ICU patients.

## Key messages

• Lp299 might be as effective as CHX in reducing the incidence of emerging potentially pathogenic bacteria in the oropharynx of intubated, mechanically ventilated, critically ill patients.

• We did not observe any adverse effects of the oral care procedure involving use of the probiotic bacterium Lp299.

## Abbreviations

APACHE II: Acute Pathophysiology and Chronic Health Evaluation; CFU: colony forming unit; CHX: chlorhexidine; CRP: C reactive protein; GI: gastrointestinal; ICU: intensive care unit; LOS: length of stay; LIS: Lung Injury Score; Lp299: *Lactobacillus plantarum *299; MRSA: methicillin-resistant *Staphylococcus aureus*; SOFA: Sequential Organ Failure Assessment; VAP: ventilator-associated pneumonia; WBC: white blood cells.

## Competing interests

Probi AB provided the study product as an unconditional grant and performed bacterial analyses. Probi AB has also done the same in earlier studies performed by BK. BJ and GM are shareholders in Probi AB, and GM resigned as a board member in 2005. Probi AB holds the patent for the investigated bacterium, but there is no patent regarding the studied application. Other financially related matters regarding GM's position as Professor at Lund University is regulated in a central and official agreement between Lund University and Probi AB.

## Authors' contributions

BK was the prime investigator and did most of the planning and performance of the study. BK handled the primary data and did most of the statistical work, and also collaborated with GM, BJ and AL to prepare and finalise the manuscript. GM contributed substantially to the analysis of the results of the bacterial cultures and completion of the manuscript. BJ took part in planning of the study and in finalising the manuscript. AL helped plan the study and was very active in preparing and competing the manuscript.
